# Neural Crest Development in Health and Disease

**DOI:** 10.3390/ijms232213684

**Published:** 2022-11-08

**Authors:** Nicolas Pilon

**Affiliations:** 1 Molecular Genetics of Development Laboratory, Department of Biological Sciences, Faculty of Sciences, Université du Québec à Montréal (UQAM), Montreal, QC H3C 3P8, Canada; pilon.nicolas@uqam.ca; 2 Centre d’Excellence en Recherche sur les Maladies Orphelines–Fondation Courtois (CERMO-FC), Université du Québec à Montréal (UQAM), Montreal, QC H2X 3Y7, Canada; 3 Department of Pediatrics, Université de Montréal, Montreal, QC H3T 1C5, Canada

The first volume of this Special Issue met its goal of covering several aspects regarding both the normal and abnormal development of neural crest cells, which form a truly unique multipotent and highly migratory cell population that only exists in vertebrates. The result is a well-balanced collection of research articles and comprehensive reviews that tackle fundamental questions about the formation, migration and multi-organ contributions of neural crest cells in diverse model organisms, while also reflecting the growing interest for applying these discoveries to understand and treat neurocristopathies in humans. These articles can be subdivided into four groups based on their links with: (*i*) early stages of global neural crest development; (*ii*) (mal)formation of upper body derivatives; (*iii*) (mal)formation of lower body derivatives; or (*iv*) neural crest-related cancer.

The first group consists of three articles addressing the questions of how neural crest cells are induced at the lateral borders of the open neural plate [1], how they collectively migrate from the closed neural tube [2], and how they stop being produced to allow formation of the roof plate in the dorsal neural tube [3]. Notably, by studying ZIC transcription factors in mouse embryos, Bellchambers et al. [1] highlight the fact that while some families of transcription factors have a broadly conserved role in neural crest specification, the identity of the involved family members may vary in mammalian vs. non-mammalian species. Grund et al. [2] investigated the role of the transmembrane protein PTK7 in migratory neural crest cells from *Xenopus* embryos, finding that it plays a key role in a cellular phenomenon known as “contact inhibition of locomotion”. In their in-depth review of the gene regulatory networks at play in the dorsal neural tube, Rekler and Kalcheim [3] discuss the possible regulatory mechanisms underlying the intriguing but currently understudied shift between neural crest and roof plate stages. 

The second group contains four articles that focus on the contribution of cranial and cardiac subpopulations of neural crest cells to craniofacial [4,5], eye [6], and heart [7] morphogenesis. Fabik et al. [5] provide a detailed review of the molecular mechanisms of craniofacial patterning and osteochondrogenesis in mouse and zebrafish embryos, also providing evidence that the morphogenesis of both mandibular and hyoid arches is governed by a shared gene regulatory network. By modeling and studying *EFTUD2* mutation-associated mandibulofacial dysostosis with microcephaly (MFDM) syndrome in mice, Beauchamp et al. [4] not only further highlight the extensive overlap between spliceosomopathies and neurocristopathies, but also unveil a new potential indirect role for P53 in alternative splicing regulation. Walker et al. [6] add to the notion that the cranial neural crest cell-derived mesenchyme around the developing eye can influence the formation of ocular structures such as the cornea, here evidencing a role for WNT and BMP signaling downstream of the transcription factor AP-2β in murine neural crest cells. Arrigo and Lin [7] review the key contribution of cardiac neural crest cells to outflow tract septation in mice through the prism of endocytic vesicle trafficking proteins, which might explain cases of congenital heart defects in humans.

In the third group of articles, one focusses on lower urinary tract innervation [8] while the other three are concerned with the largest branch of the peripheral nervous system: the enteric nervous system [9,10,11]. In Ritter et al. [8], type 3 serotonin receptor signaling is identified as a new regulator of neurogenesis in sacral neural crest cell-derived pelvic ganglia in mice, a finding of potential clinical relevance for the control of bladder function in humans. Ji et al. [9] summarize current views about the role of the microenvironment around neural crest-derived enteric neural progenitors in modulating the development of the enteric nervous system and a related pathological condition known as Hirschsprung disease. Rueckert and Ganz [10] extend the discussion to the capacity of the enteric nervous system to regenerate from neural crest-derived tissue-resident stem cells, further advocating that the zebrafish is ideally suited for these studies. The study from Soret et al. [11] directly relates to both reviews mentioned above, highlighting that Hirschsprung-like disease caused by microenvironment perturbation in mice can be rescued by the GDNF-induced regeneration of the enteric nervous system, while also showing that the enteric nervous system is more sensitive to genetic background changes than other neural crest derivatives.

Last but not least, Mohamad et al. remind us that the abnormal development of neural crest cells can not only lead to congenital malformations, but also cancer, as reviewed here for malignant peripheral nerve sheath tumors that arise from neural crest-derived Schwann cells [12].
